# Supplementary material for: Nanoscale zero-field electron spin resonance spectroscopy
Source: Nat Commun. 2018 Apr 19;9:1563. doi: 10.1038/s41467-018-03969-4 (PMC5908811; doi:10.1038/s41467-018-03969-4)
Supplement: Supplementary file 1 — Supplementary Information [file 41467_2018_3969_MOESM1_ESM.pdf]

Supplementary Information

Nanoscale zero-field electron spin resonance spectroscopy

Kong *et al.*

Supplementary Note 1. DESCRIPTION OF THE NV-P1 SYSTEM

Theoretical Model of the NV-P1 system

The full Hamiltonian of a NV center coupled to a P1 center is

$$\begin{aligned} H_{\mathrm{T}} &= H_{\mathrm{NV}} + H_{\mathrm{P1}} + H_{\mathrm{int}}, \\ H_{\mathrm{NV}} &= D S_z^{\mathrm{NV}2}, \\ H_{\mathrm{P1}} &= \mathbf{S}^{\mathrm{P1}} \cdot \mathbb{A} \cdot \mathbf{I}, \\ H_{\mathrm{int}} &= \frac{\mu_0 \gamma_{\mathrm{NV}} \gamma_{\mathrm{P1}} \hbar}{4\pi} [\frac{\mathbf{S}^{\mathrm{NV}} \cdot \mathbf{S}^{\mathrm{P1}}}{r^3} - \frac{3(\mathbf{S}^{\mathrm{NV}} \cdot \mathbf{r})(\mathbf{S}^{\mathrm{P1}} \cdot \mathbf{r})}{r^5}], \end{aligned} \tag{1}$$

where $\mathbf{S}^{\mathrm{NV}}$, $\mathbf{S}^{\mathrm{P1}}$ and $\mathbf{I}$ are the spin operators for the NV electron spin, the P1 electron spin and the P1 nuclear spin, respectively, $D = 2\pi \times 2.87$ GHz is the zero-field splitting of the NV center, $\mathbb{A}$ is the hyperfine interaction between the P1 electron spin and nuclear spin, $\mathbf{r}$ is the separation vector between the NV and P1 centers. If a resonant microwave is applied with the form $H_1 = \Omega \cos Dt S_x^{\mathrm{NV}}$, the self Hamiltonian of the NV center in the rotating reference frame becomes

$$H_{\mathrm{NV}}^{\mathrm{rot}} = \frac{\Omega}{2} S_x^{\mathrm{NV}}, \tag{2}$$

and the Hamiltonian of the interaction between the NV center and the P1 centers can be simplified to

$$H_{\mathrm{int}} = \frac{\mu_0 \gamma_{\mathrm{NV}} \gamma_{\mathrm{P1}} \hbar}{4\pi r^3} [S_z^{\mathrm{NV}} S_z^{\mathrm{P1}} - 3 S_z^{\mathrm{NV}} (\hat{r} \cdot \vec{e}_z)(\mathbf{S}^{\mathrm{P1}} \cdot \hat{r})]. \tag{3}$$

The presence of other bath spins will induce relaxation of both the NV and P1 centers, which can be modeled by the following master equation

$$\frac{d\rho}{dt} = -i[H_{\mathrm{T}}, \rho] + \sum_{j=1}^{2} \mathcal{L}_j \rho \mathcal{L}_j^\dagger - \frac{1}{2}(\mathcal{L}_j^\dagger L_j \rho + \rho \mathcal{L}_j^\dagger L_j), \tag{4}$$

where $\mathcal{L}_1 = \sqrt{2\Gamma_{\mathrm{NV}}} S_z^{\mathrm{NV}}$ and $\mathcal{L}_2 = \sqrt{2\Gamma_{\mathrm{P1}}} S_z^{\mathrm{P1}}$ are the Lindbladian operators corresponding to the transverse relaxation of the NV and P1 center respectively, with $\Gamma_{\mathrm{NV}} = 1/T_{1\rho}$ and $\Gamma_{\mathrm{P1}} = 1/T_2^*$. Here the longitudinal relaxation is ignored as it is much slower than the transverse relaxation.

Analytical solution

To analytically describe the evolution of the NV center, here we deal with the two kinds of relaxation separately. **Transverse relaxation of the NV center.** In the far off-resonance case, the interaction item H_{int} can be ignored. If the initial state is an eigenstate of $H_{\mathrm{NV}}^{\mathrm{rot}}$, for example, $|-1\rangle_{\mathrm{d}}$ (formula Eq. 1 in the main text), and we only concern the populations of the dressed states, then the solution of Supplementary Eq. 4 is given by

$$\begin{aligned} P^0_{|-1\rangle_{\mathrm{d}}}(t) &= \frac{1}{3} + \frac{1}{6} e^{-3\Gamma_{\mathrm{NV}} t} + \frac{1}{2} e^{-\Gamma_{\mathrm{NV}} t}, \\ P^0_{|0\rangle_{\mathrm{d}}}(t) &= \frac{1}{3} - \frac{1}{3} e^{-3\Gamma_{\mathrm{NV}} t}, \\ P^0_{|1\rangle_{\mathrm{d}}}(t) &= \frac{1}{3} + \frac{1}{6} e^{-3\Gamma_{\mathrm{NV}} t} - \frac{1}{2} e^{-\Gamma_{\mathrm{NV}} t}. \end{aligned} \tag{5}$$

Transverse relaxation of the P1 center. In the case of strong relaxation of P1 centers and relative weak interaction with the NV center, the coupling of P1 centers to the NV center can be treated as a semiclassical oscillatory field [1]

$$H_{\mathrm{int}} : \mathcal{B}(\Delta\omega_{ij}) e^{i\Delta\omega_{ij} t} + \mathcal{B}^\dagger(\Delta\omega_{ij}) e^{-i\Delta\omega_{ij} t}, \tag{6}$$

where ω_{ij} is the energy level splitting of P1 centers and $\mathcal{B}(\Delta\omega_{ij})$ is the dipolar coupling strength. If the initial state is $|-1\rangle_{\mathrm{d}}$, then the solution of Supplementary Eq. 4 is given by

$$\begin{aligned} P^1_{|-1\rangle_{\mathrm{d}}}(t) &= \frac{1}{3} + \frac{1}{6}e^{-3\gamma t} + \frac{1}{2}e^{-\gamma t}, \\ P^1_{|0\rangle_{\mathrm{d}}}(t) &= \frac{1}{3} - \frac{1}{3}e^{-3\gamma t}, \\ P^1_{|1\rangle_{\mathrm{d}}}(t) &= \frac{1}{3} + \frac{1}{6}e^{-3\gamma t} - \frac{1}{2}e^{-\gamma t}. \end{aligned} \tag{7}$$

where $\gamma = \frac{1}{8}\sum_{i<j}\frac{b_{ij}^2\Gamma_{\mathrm{P1}}}{(\Omega-2\Delta\omega_{ij})^2+\Gamma_{\mathrm{P1}}^2}$, $b_{ij} \equiv \sqrt{\langle\mathcal{B}(\Delta\omega_{ij})^2\rangle}$. Note here the solution has the same form with Supplementary Eq. 5, as the cross-polarization process will also induce the relaxation of the NV center.

By combining Supplementary Eq. 5 and Supplementary Eq. 7 together, the complete solution of Supplementary Eq. 4 is given by

$$\begin{aligned} P_{|-1\rangle_{\mathrm{d}}}(t) &= \frac{1}{3} + \frac{1}{6}e^{-3(\Gamma_{\mathrm{NV}}+\gamma)t} + \frac{1}{2}e^{-(\Gamma_{\mathrm{NV}}+\gamma)t}, \\ P_{|0\rangle_{\mathrm{d}}}(t) &= \frac{1}{3} - \frac{1}{3}e^{-3(\Gamma_{\mathrm{NV}}+\gamma)t}, \\ P_{|1\rangle_{\mathrm{d}}}(t) &= \frac{1}{3} + \frac{1}{6}e^{-3(\Gamma_{\mathrm{NV}}+\gamma)t} - \frac{1}{2}e^{-(\Gamma_{\mathrm{NV}}+\gamma)t}. \end{aligned} \tag{8}$$

In the short time limit (i.e., $(\Gamma_{\mathrm{NV}}+\gamma)t \ll 1$), we have

$$P_{|-1\rangle_{\mathrm{d}}}(t) \approx 1 - \Gamma_{\mathrm{NV}}t - \gamma t. \tag{9}$$

To determine b_{ij}, let's consider the items in the Supplementary Eq. 3. Here the coordinate system is chosen with the z axis towards the N-V axis, and the directions of separation vector $\mathbf{r}$ and the principal axis of P1 are characterized by θ_r, ϕ_r and $\theta_{\mathrm{e}}, \phi_{\mathrm{e}}$, respectively. In this frame, Supplementary Eq. 3 can be rewrite as

$$H_{\mathrm{int}} = S_z^{\mathrm{NV}}(b_{zx}S_x^{\mathrm{P1}} + b_{zy}S_y^{\mathrm{P1}} + b_{zz}S_z^{\mathrm{P1}}), \tag{10}$$

with components $b_{zj} = \frac{\mu_0\gamma_{\mathrm{NV}}\gamma_{\mathrm{P1}}\hbar}{4\pi r^3}\cdot\eta_{zj}, j = x, y, z$, where

$$\begin{aligned} \eta_{zx} &= -3\sin\theta_r\cos\phi_r\cos\theta_r, \\ \eta_{zy} &= -3\sin\theta_r\sin\phi_r\cos\theta_r, \\ \eta_{zz} &= 1 - 3\cos^2\theta_r. \end{aligned} \tag{11}$$

These components corresponds to the six allowed magnetic dipole transitions given by Eq. 7 in the main text, as each of the transitions is linearly polarized in the x, or y or z direction [2]:

$$\begin{aligned} \Delta\omega_{12} &= \frac{1}{2}|A_{xx}+A_{yy}|, \Delta\omega_{34} = \frac{1}{2}|A_{xx}-A_{yy}| : z-\text{polarized} \\ \Delta\omega_{13} &= \frac{1}{2}|A_{yy}+A_{zz}|, \Delta\omega_{24} = \frac{1}{2}|A_{yy}-A_{zz}| : x-\text{polarized} \\ \Delta\omega_{14} &= \frac{1}{2}|A_{xx}+A_{zz}|, \Delta\omega_{23} = \frac{1}{2}|A_{xx}-A_{zz}| : y-\text{polarized} \end{aligned} \tag{12}$$

Note here the x, y and z is defined in the P1 principal axis frame, where the hyperfine tensor $\mathbb{A}$ is diagonal. In the NV frame, the hyperfine tensor $\mathbb{A}$ can be written as

$$\mathbb{A} = \mathbb{R}\cdot\begin{pmatrix} A_{xx} & & \\ & A_{yy} & \\ & & A_{zz} \end{pmatrix}\cdot\mathbb{R}^{-1}, \tag{13}$$

where

$$\mathbb{R} = \begin{pmatrix} \cos\theta_{\mathrm{e}}\cos\phi_{\mathrm{e}} & \cos\theta_{\mathrm{e}}\sin\phi_{\mathrm{e}} & -\sin\theta_{\mathrm{e}} \\ -\sin\phi_{\mathrm{e}} & \cos\phi_{\mathrm{e}} & 0 \\ \sin\theta_{\mathrm{e}}\cos\phi_{\mathrm{e}} & \sin\theta_{\mathrm{e}}\sin\phi_{\mathrm{e}} & \cos\theta_{\mathrm{e}} \end{pmatrix} \tag{14}$$

is the rotational transform from the P1 frame to the NV frame. Hence the actual coefficients are

$$\left(\eta_{zx}^{'}, \eta_{zy}^{'}, \eta_{zz}^{'} \right) = \left(\eta_{zx}, \eta_{zy}, \eta_{zz} \right) \cdot \mathbb{R}. \tag{15}$$

For the case of axial symmetric $\mathbb{A}$, $\eta_{z\perp}^{'} = \sqrt{\eta_{zx}^{'2} + \eta_{zy}^{'2}}$.

To verify the above analytical expressions, a numerical calculation according to Supplementary Eq. 4 without any assumptions is also given. As shown in Supplementary Fig. **1**, the analytical calculations show good agreement with the numerical calculations when $\Gamma_{\mathrm{P1}} \gtrsim 17b$. For the experiment in this work, the analytical expression reliable if the NV-P1 spacing is larger than 8 nm.

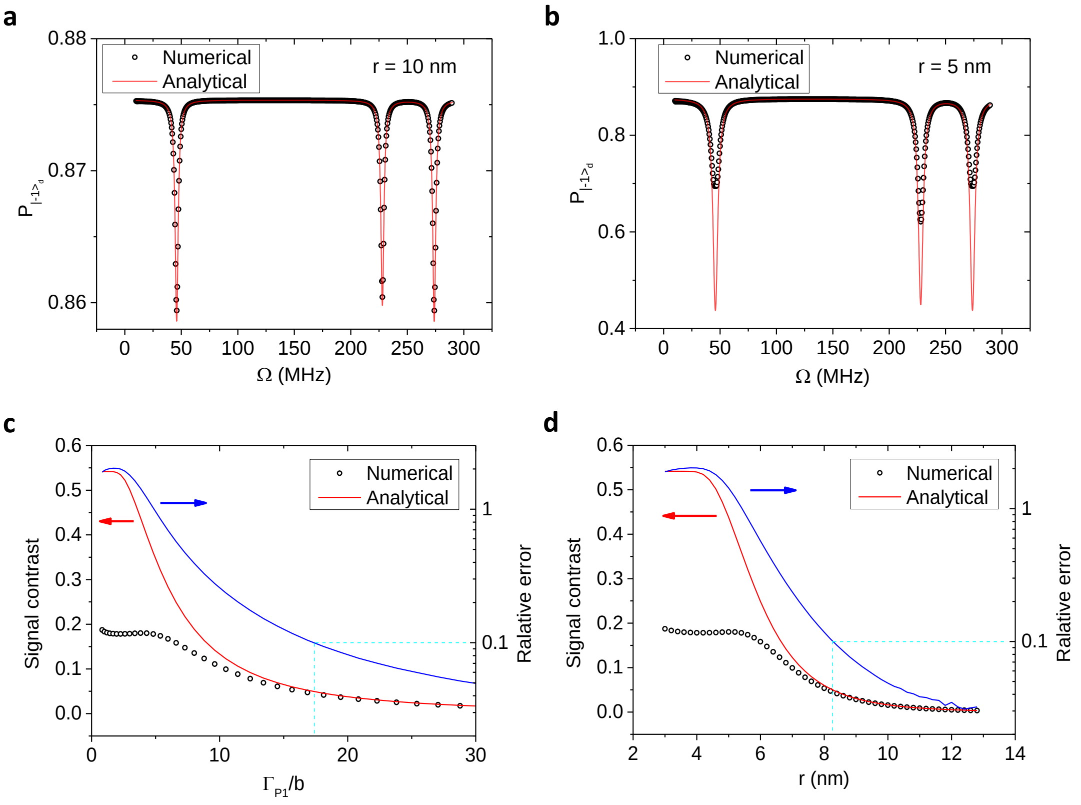

Supplementary Figure 1: **Comparison of the analytical and numerical calculations.** (**a**)(**b**) Simulated ZF-ESR spectrum of a P1 center detected by a NV center with NV-P1 spacing of **a**, $r = 10$ nm and **b**, $r = 5$ nm. Other parameters used in the simulation are $\theta_{\mathrm{e}} = 0$, $\phi_{\mathrm{e}} = 0$, $\theta_r = \pi/6$, $\phi_r = 0$, $t = 10$ μs, $T_{1\rho} = 70$ μs, and $\Gamma_{\mathrm{P1}} = 10$ MHz. (**c**) Calculated signal contrast of the right peak with different NV-P1 spacing r. Blue line is the relative error of analytical calculation given by $|\mathrm{Ana} - \mathrm{Num}|/\mathrm{Num}$. The analytical calculation shows good agreement with the numerical one when $r \gtrsim 8$ nm. (**d**) The same data in **c** but the x axis is given by the ratio of the relaxation rate of P1 and the coupling rate. The analytical calculation shows good agreement with the numerical one when $\Gamma_{\mathrm{P1}} \gtrsim 17b$.

Interaction with multiple P1 centers

Considering that the NV center is coupled to many P1 centers, the sum of signal is induced by the total dipolar coupling variance $\sum_k b_k^2$. Since the NV and P1 centers are created by ion implantations in the experiment, all the defects are concentrated in a thin (several nanometers) layer. Supposing there is a circle of P1 centers with NV located at the center, as shown in Supplementary Fig. **2**. In the lab frame (with the NV center towards the 111 direction), the direction of separation vector $\mathbf{r}$ is

$$\hat{r}_{\mathrm{lab}} = (\cos\phi, \sin\phi, 0), \tag{16}$$

which can be transform to the NV frame by

$$\hat{r}_{\mathrm{NV}} = \hat{r}_{\mathrm{lab}} \cdot \mathbb{T}, \tag{17}$$

with

$$\mathbb{T} = \begin{pmatrix} \sqrt{\frac{2}{3}} & 0 & \sqrt{\frac{1}{3}} \\ -\sqrt{\frac{1}{6}} & \sqrt{\frac{1}{2}} & \sqrt{\frac{1}{3}} \\ -\sqrt{\frac{1}{6}} & -\sqrt{\frac{1}{2}} & \sqrt{\frac{1}{3}} \end{pmatrix}. \tag{18}$$

Then the three dipolar coupling coefficients are

$$\begin{aligned} \eta_{zx} &= -\frac{1}{2\sqrt{2}}[1 + \sin 2\phi + 3\cos 2\phi], \\ \eta_{zy} &= -\sqrt{\frac{3}{2}}\sin\phi[\sin\phi + \cos\phi], \\ \eta_{zx} &= -\sin 2\phi. \end{aligned} \tag{19}$$

After transformed to the P1 frame according to Supplementary Eq. 15, the mean variance can be calculated by

$$\langle \eta_{zj}^{'2} \rangle = \frac{1}{2\pi}\int_0^{2\pi} \eta_{zj}^{'2} d\phi, j = x, y, z. \tag{20}$$

For the four kinds of orientations of P1 centers, the calculated values are given in Supplementary Table **1**. As the probabilities of P1 towards these directions are equal, the mean variance are $\langle \eta_{z\perp}^{'2} \rangle = 5/4$ and $\langle \eta_{zz}^{'2} \rangle = 3/4$, corresponding to the intensity of middle and left/right peak in Fig. 3c the main text. Therefore, according to Supplementary Eq. 9, the average signal of a P1 center detected by a NV center with NV-P1 spacing r is given by

$$\begin{aligned} \bar{S}(r,t) &= P^{\text{off}}_{|-1\rangle_{\text{d}}}(t) - P^{\text{on}}_{|-1\rangle_{\text{d}}}(t) \\ &= \left(\frac{\mu_0 \gamma_{\text{NV}} \gamma_{\text{P1}} \hbar}{4\pi}\right)^2 \cdot \frac{\langle \eta^2 \rangle t}{8\Gamma_{\text{P1}} r^6}, \end{aligned} \tag{21}$$

where 'off' and 'on' denote far off-resonance $|\Omega - 2\Delta\omega_{ij}| \gg \Gamma_{\text{P1}}$ and on-resonance cases $\Omega = 2\Delta\omega_{ij}$, respectively. The signal of multiple P1 centers can be given by $\sum_r \bar{S}(r,t)$.

Supplementary Table 1: Averaged dipolar coupling variance.

$(\theta_\text{e}, \phi_\text{e})$	$\langle \eta_{zx}^{'2} \rangle$	$\langle \eta_{zy}^{'2} \rangle$	$\langle \eta_{zz}^{'2} \rangle$
$(0, 0)$	$\frac{3}{4}$	$\frac{3}{4}$	$\frac{1}{2}$
$(\arccos(-\frac{1}{3}), 0)$	$\frac{5}{12}$	$\frac{3}{4}$	$\frac{5}{6}$
$(\arccos(-\frac{1}{3}), \frac{2\pi}{3})$	$\frac{11}{12}$	$\frac{1}{4}$	$\frac{5}{6}$
$(\arccos(-\frac{1}{3}), -\frac{2\pi}{3})$	$\frac{5}{12}$	$\frac{3}{4}$	$\frac{5}{6}$

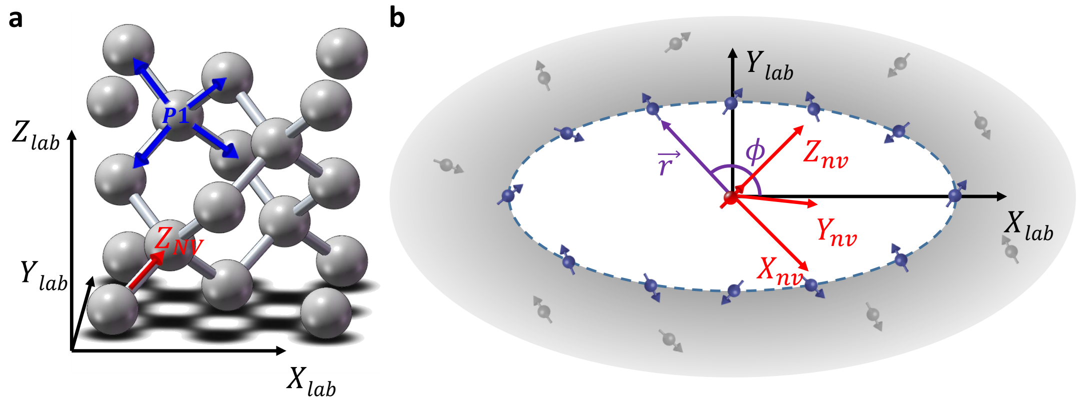

Supplementary Figure 2: **A single NV center coupled to multiple P1 centers.** (**a**) Schematic diagram of the lab, NV and P1 frame. (**b**) Locations of P1 centers with respect to the central NV center.

Supplementary Note 2. DOUBLE ELECTRON-ELECTRON RESONANCE SPECTRUM

Double electron-electron resonance (DEER) spectrum was measured to confirm the existence of P1 centers. As shown in Supplementary Fig. **3**, the four side peaks indicate the signal of ^{15}N P1 centers, while the two central peaks indicate the signal of background spins. As discuss in the main text, there exists dense dangling bonds on the diamond surface with short correlation time, they are likely to be the source of the broad peak. Since the diamond is implanted with high dose of ion, there exists other vacancy defects inside the diamond. They probably induce the narrow background peak, due to the relative longer correlation time.

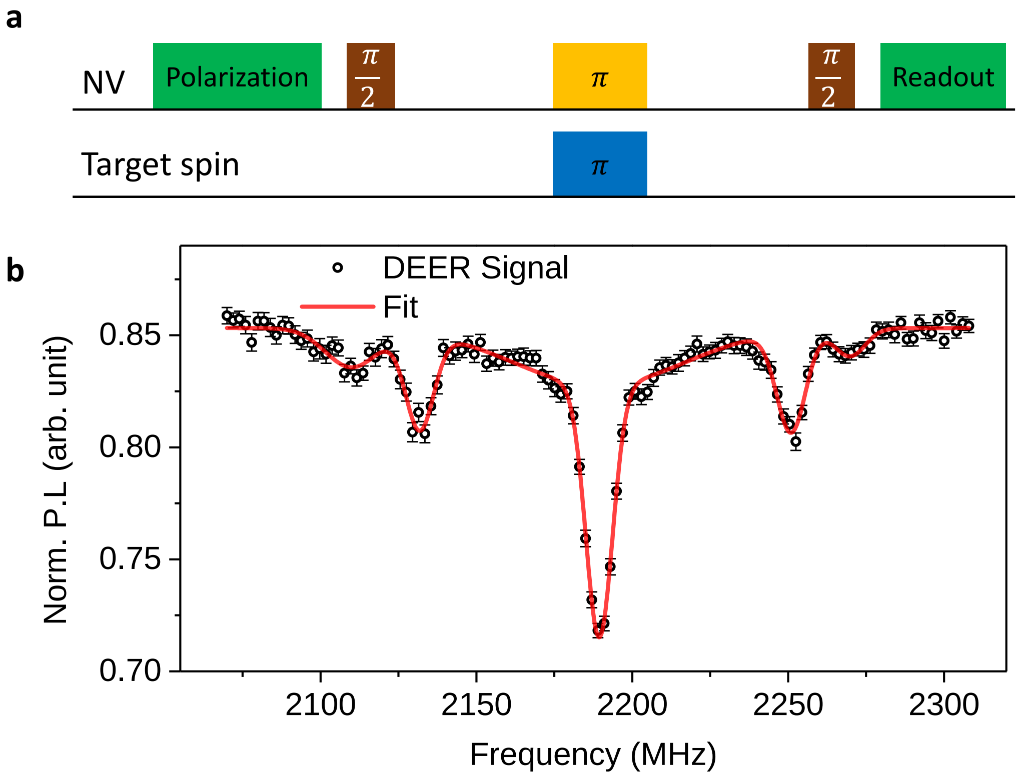

Supplementary Figure 3: **DEER spectrum of ^{15}N P1 center.** (**a**) Pulse sequence for DEER measurement. The frequency of the pulse applied on the target spin (blue box) is swept. (**b**) Measured DEER spectrum. The measurement was perform in a magnetic field of 781 Gauss. The circle points are experimental results while the solid line is a six-Gaussian-peak fitting. Note the central line contains a narrow and a broad peak with the same frequency. Error bars indicate ±1 standard error of the mean (s.e.m). The measurement is repeated 13.7 million times.

Supplementary Note 3. CALIBRATION OF THE BASELINE OF ZERO-FIELD ESR SPECTRUM

As mentioned in the main text, the spin-locking relaxation time $T_{1\rho}$ of NV centers depends on the driving power. Supplementary Fig. **4** shows that $T_{1\rho}$ first increases and then decreases with the increasing driving power, which is similar with a previous reported work [3]. In low-power region, the spin-locking relaxation of NV center is dominated by its coupling with the fluctuated bath spins, which induces Lorentz-type noise central at zero in zero magnetic field [4]. Note in the above section, we have observed these bath spins in the DEER spectrum. Here the narrow peak is not observed, and the broad low-frequency background signal can be removed after calibration. After careful chemical treatment of the diamond surface, the density of the dangling bonds can be further reduced, then this low-frequency background signal can be narrowed and naturally removed. With the increasing driving power, the driving error grows proportionally, which will dominate the spin-locking relaxation in high-power region. The driving error is likely obeying normal distribution, as the baseline of Supplementary Fig. **4** in high-power region can be fitted by a Gaussian lineshape decay very well. Fig. 3c in the main text was obtained by eliminated the baseline in Supplementary Fig. **4**.

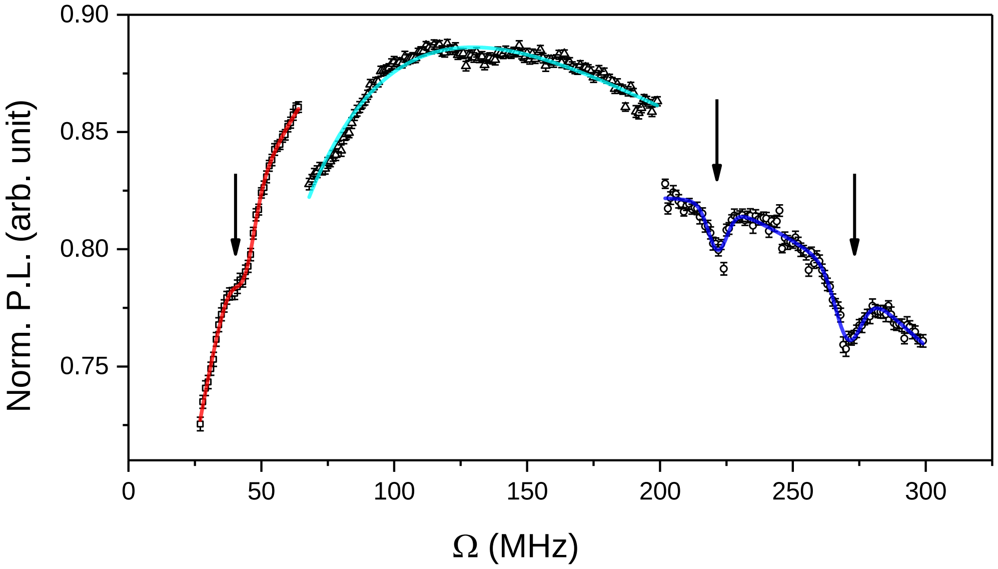

Supplementary Figure 4: **Zero-field ESR spectrum of ^{15}N P1 center without baseline calibration.** The whole spectrum was acquired by three sequential measurements. In low-power region, the data (square points) was fitted by a Gaussian peak with a Lorentz lineshape decay baseline (red curve). In medium-power region , the data (triangle points) was fitted by Lorentz lineshape decay plus Gaussian lineshape decay (turquoise curve). In high-power region, the data (circle points) was fitted by two Gaussian peaks with a Gaussian lineshape decay baseline (blue curve). The three arrows denote the three transition frequencies of the P1 center given in the main text. Error bars indicate ± 1 s.e.m. The measurement is repeated 5, 5 and 2 million times for the low-power, medium-power and high-power data, respectively.

Supplementary Note 4. EXPERIMENTAL IMPERFECTIONS

Driving power fluctuation

The driving power was not stable due to the electric noise in the microwave circuits. To evaluate the power fluctuations, the measurement of zero-field ESR spectra was divided into many rounds, each lasted for ~ 3 minutes. Before every round, a Rabi oscillation with constant input microwave was measured to record the driving power (i.e. the Rabi frequency). As shown in Supplementary Fig. **5**a, the driving power had fast fluctuations and slow drifts. The slow drifts can be eliminated by calibrating the input power in each experimental rounds. The fast fluctuations were characterized by the relative power differences of each neighboring rounds, as shown in Supplementary Fig. **5**a, which indicated the fluctuation is $< 0.5\%$.

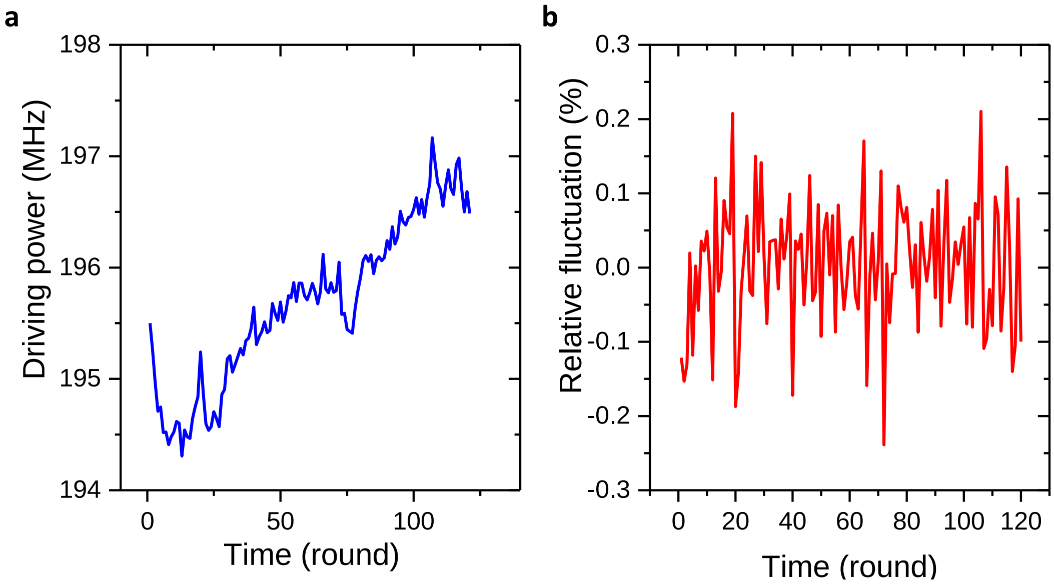

Supplementary Figure 5: **Fluctuation of the driving power during the experiment.** (**a**)Recorded driving power in each experiment round. (**b**)Relative fluctuation calculated by the relative power differences of each neighboring rounds in (a).

Residual magnetic field

In the presence of magnetic field, the Hamiltonian of P1 center (Eq. 3 in the main text) is modified to

$$H_0 = \gamma_e \mathbf{B} \cdot \mathbf{S} + \mathbf{S} \cdot \mathbb{A} \cdot \mathbf{I}, \tag{22}$$

where an extra Zeeman energy $\gamma_e \mathbf{B} \cdot \mathbf{S}$ is added, here $\mathbf{B}$ is the residual magnetic field, which is mainly contributed by the geomagnetic field with magnitude ~ 0.5 G. As the Zeeman energy is much smaller than the hyperfine interaction, only the component paralleling to the P1 principal axis remains, which induces an energy splitting $\gamma_e \mathbf{B}_{\|}$ of electron spin. The diamond was placed such that the geomagnetic field was nearly towards to the 110 direction. Then the angle between the magnetic field and P1 centers was either $\sim 90°$ or $\sim 35.3°$. Supplementary Fig. **6** gives the numerically calculated zero-field ESR spectrum with different magnetic field magnitude. The left and right peak splitting induced by the geomagnetic field are ~ 2.3 MHz, which only leads to peak broadening of the spectrum in Fig. 3c in the main text. While the middle peak is insensitive to the magnetic field, which is a possible reason of that the left and right peaks in Fig. 3c in the main text are slightly broader than the middle peak.

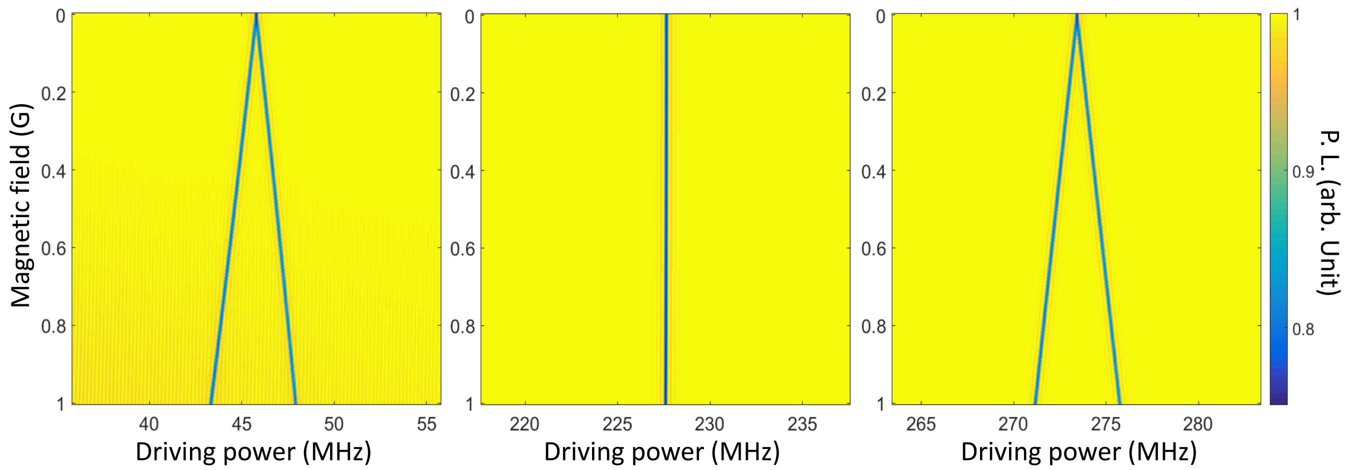

Supplementary Figure 6: **Numerically calculated zero-field ESR spctra of ^{15}N P1 centers in magnetic field with different magnitudes.** Here the angle between the magnetic field and the P1 direction is $35.3°$.

Supplementary Note 5. ZERO-FIELD ESR SPECTRUM OF ^{14}N P1 CENTER

The Hamiltonian of ^{15}N P1 center has been given by Eq. 3 in the main text. For ^{14}N P1 center, an additional nuclear quadrupole coupling need to be taken into account, and the Hamiltonian is given by

$$H_{\mathrm{P1}} = \mathbf{S} \cdot \mathbb{A} \cdot \mathbf{I} + \mathbf{I} \cdot \mathbb{Q} \cdot \mathbf{I}, \tag{23}$$

where $\mathbf{S}$ ($S = 1/2$) and $\mathbf{I}$ ($I = 1$) are the operators for the electron and nuclear spin respectively, $\mathbb{A}$ is the hyperfine tensor, and $\mathbb{Q}$ is the nuclear quadrupole coupling tensor. In the principal axis (N-C axis) system (PAS), both the $\mathbb{A}$ and $\mathbb{Q}$ are diagonal

$$\mathbb{A} = \begin{pmatrix} A_{xx} & & \\ & A_{yy} & \\ & & A_{zz} \end{pmatrix}, \mathbb{Q} = \begin{pmatrix} V_{xx} & & \\ & V_{yy} & \\ & & V_{zz} \end{pmatrix} \tag{24}$$

with $A_{xx} = A_{yy} = A_{\perp} = 81.3$ MHz, $A_{zz} = A_{\parallel} = 114$ MHz [5], $|V_{zz}| > |V_{xx}| = |V_{yy}|$, and $V_{xx} + V_{yy} + V_{zz} = 0$ [6]. Then the Hamiltonian of ^{14}N P1 center becomes

$$H_{\mathrm{P1}}^{\mathrm{PAS}} = A_{\parallel} S_z I_z + A_{\perp}(S_x I_x + S_y I_y) + Q[I_z^2 - \frac{1}{3} I(I+1)], \tag{25}$$

where $Q = \frac{3}{2} V_{zz} = -4.2$ MHz [7]. The corresponding eigenstates are

$$\begin{aligned} |\phi_1\rangle &= \sin\alpha |\uparrow, 0\rangle - \cos\alpha |\downarrow, 1\rangle, \\ |\phi_2\rangle &= \sin\alpha |\downarrow, 0\rangle - \cos\alpha |\uparrow, -1\rangle, \\ |\phi_3\rangle &= \cos\alpha |\uparrow, 0\rangle + \sin\alpha |\downarrow, 1\rangle, \\ |\phi_4\rangle &= \cos\alpha |\downarrow, 0\rangle + \sin\alpha |\uparrow, -1\rangle, \\ |\phi_5\rangle &= |\uparrow, 1\rangle, \\ |\phi_6\rangle &= |\downarrow, -1\rangle, \end{aligned} \tag{26}$$

where $|\uparrow\rangle$ and $|\downarrow\rangle$ denote the electron spin states while $|1\rangle$, $|0\rangle$ and $|-1\rangle$ denote the nuclear spin states, and $\alpha = \arctan\{[\sqrt{8A_{\perp}^2 + (A_{\parallel} - 2Q)^2} - A_{\parallel} + 2Q]/\sqrt{8}A_{\perp}\}$. The corresponding six eigenenergies are

$$\begin{aligned} \omega_1 = \omega_2 &= -\frac{1}{4}A_{\parallel} - \frac{1}{6}Q + \frac{1}{4}\sqrt{8A_{\perp}^2 + (A_{\parallel} - 2Q)^2}, \\ \omega_3 = \omega_4 &= -\frac{1}{4}A_{\parallel} - \frac{1}{6}Q - \frac{1}{4}\sqrt{8A_{\perp}^2 + (A_{\parallel} - 2Q)^2}, \\ \omega_5 = \omega_6 &= \frac{1}{2}A_{\parallel} + \frac{1}{3}Q. \end{aligned} \tag{27}$$

Supplementary Fig. **7**a illustrates the transitions between different energy levels. Due to the energy level degeneracy, only three kinds of transition frequencies are expected with values

$$\begin{aligned} \Delta\omega_{13} = \Delta\omega_{24} &= \frac{1}{2}\sqrt{8A_{\perp}^2 + (A_{\parallel} - 2Q)^2}, \\ \Delta\omega_{35} = \Delta\omega_{46} &= \frac{3}{4}A_{\parallel} + \frac{1}{2}Q - \frac{1}{4}\sqrt{8A_{\perp}^2 + (A_{\parallel} - 2Q)^2}, \\ \Delta\omega_{15} = \Delta\omega_{26} &= \frac{3}{4}A_{\parallel} + \frac{1}{2}Q + \frac{1}{4}\sqrt{8A_{\perp}^2 + (A_{\parallel} - 2Q)^2}. \end{aligned} \tag{28}$$

The calculated values are 130.25 MHz, 18.28 MHz, and 148.52 MHz, respectively.

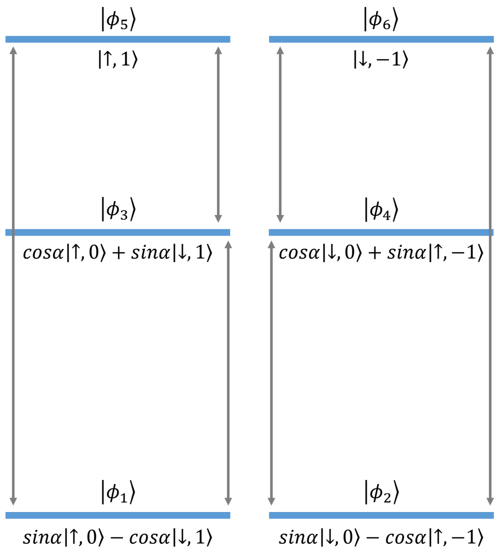

Supplementary Figure 7: **Energy levels of ^{14}N P1 center and transitions between them.** The eigenstates $|\phi_1\rangle$ to $|\phi_6\rangle$ are given by the linear combination of the eigenstates of the electron spin and the nuclear spin (see the formula below the bold line). There exists energy level degeneracy due to the axially symmetric hyperfine interaction.

The zero-field ESR spectrum of ^{14}N P1 centers was measured on another diamond, which was implanted using 8 keV $^{14}N_2^+$ ions with dosage of 1×10^{13} cm^{-2}. Using the same method presented in the main text, a fingerprint-like spectrum was acquired, as illustrated in Supplementary Fig. **8**, with three obvious peaks at 38.9 ± 3.7 MHz, 262.0 ± 7.5 MHz and 298.7 ± 5.4 MHz, which consisted with the above calculation results (note that $\Omega = 2\Delta\omega_{ij}$).

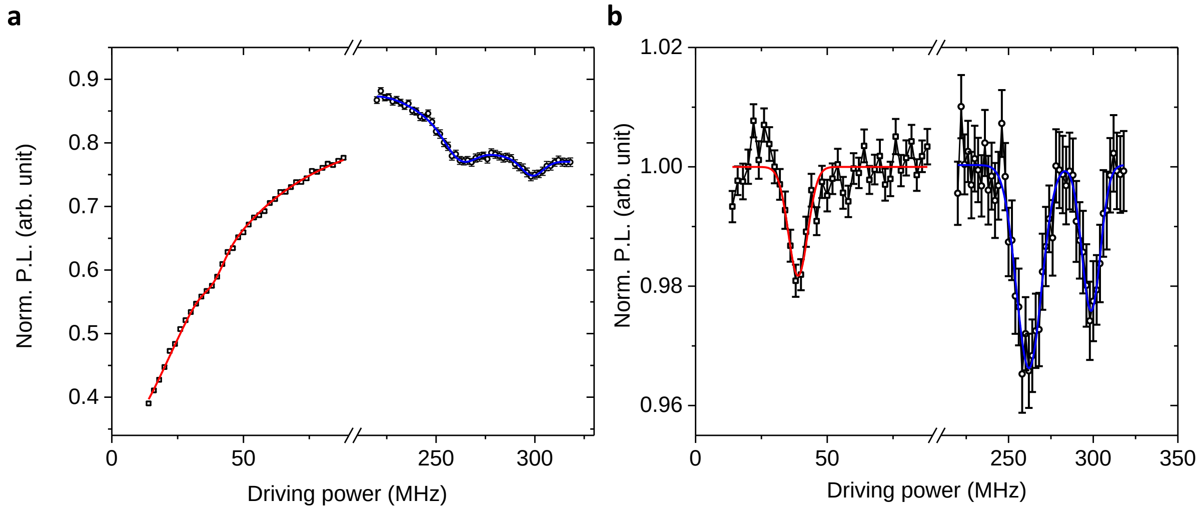

Supplementary Figure 8: **Measured zero-field ESR spectrum of ^{14}N P1 center.** (**a**)Orignal spectrum without baseline calibration. In low-power region, the data (square points) was fitted by a Gaussian peak with a Lorentz lineshape decay baseline (red curve). In high-power region, the data (circle points) was fitted by two Gaussian peaks with a Gaussian lineshape decay baseline (blue curve). Error bars indicate ± 1 s.e.m. The measurement is repeated 5 and 1 million times for the low-power and high-power data, respectively. (**b**)Baseline calibrated spectrum.

Supplementary References

[1] Hall, L. T. *et al.* Detection of nanoscale electron spin resonance spectra demonstrated using nitrogen-vacancy centre probes in diamond. *Nat. Commun.* **7**, 10211 (2016).

[2] McConnell, H. M., Thompson, D. D. & Fessenden, R. W. Hyperfine absorption spectrum of $CH(COOH)_2^*$. *Proc. Natl. Acad. Sci.* **45**, 1600–1601 (1959).

[3] Xu, X. *et al.* Coherence-protected quantum gate by continuous dynamical decoupling in diamond. *Phys. Rev. Lett.* **109**, 070502 (2012).

[4] Jarmola, A., Acosta, V. M., Jensen, K., Chemerisov, S. & Budker, D. Temperature- and magnetic-field-dependent longitudinal spin relaxation in nitrogen-vacancy ensembles in diamond. *Phys. Rev. Lett.* **108**, 197601 (2012).

[5] Smith, W. V., Sorokin, P. P., Gelles, I. L. & Lasher, G. J. Electron-spin resonance of nitrogen donors in diamond. *Phys. Rev.* **115**, 1546–1552 (1959).

[6] Gready, J. E. The relationship between nuclear quadrupole coupling constants and the asymmetry parameter. the interplay of theory and experiment. *J. Am. Chem. Soc.* **103**, 3682–3691 (1981).

[7] de Lange, G. *et al.* Controlling the quantum dynamics of a mesoscopic spin bath in diamond. *Sci. Rep.* **2**, 382 (2012).
